# Improved efficiency and diagnostic utility of inpatient transthoracic echocardiography following implementation of a sonographer-initiated perflutren-based contrast administration protocol

**DOI:** 10.1186/s12947-020-00215-0

**Published:** 2020-08-17

**Authors:** Ryan Prentice, Homayoun Ahmadian, Dustin Thomas, Jeremy Berger, Rosco Gore

**Affiliations:** Cardiology Division, Brooke Army Medical Center, San Antonio, TX USA

**Keywords:** Contrast, Contrast Echo, Sonographer, Echocardiogram, Ultrasound enhancing agent

## Abstract

**Background:**

Up to 20% of resting echocardiograms obtained are suboptimal leading to further downstream testing and delays in diagnosis. Contrast enhanced echocardiography is well established and endorsed for use by the American Society of Echocardiography (ASE) in clinical scenarios when 2 or more adjacent wall segments are not well visualized; however, varied institutional protocols and practices in place limit such use due to increased time and personnel needed to obtain such imaging.

**Methods:**

The purpose of this study was to determineif sonographer administered echo contrast led to decreased time to complete inpatient echocardiography exams when compared to the current institutional policy of having a registered nurse perform administration of contrast via a case-control approach. Sonographers received a one-day training course on the techniques for contrast administration. Baseline completion times (time from 1st image to last image) were reviewed in studies from March 2015 to May 2015. Sonographers who received training began self-administration of contrast the first week of June 2015. After a familiarization period, study completion times were recorded from September 2015 to December 2015 and compared to those during the baseline phase. Sonographers were not informed that they were being monitored. Patients and the public were not involved in the design or conduct of our study.

**Results:**

A total of 320 patients were included for analysis. Time spent obtaining contrast enhanced imaging was not significant between the two groups (*p* = 0.67). Time spent to complete each echocardiogram (time from first echocardiogram image to the last contrast enhanced echocardiogram image) was significant between the two groups (37.5 ± 10.9 min sonographer administered v 49.6 ± 12.5 min in nurse administered group, *p* < 0.001).

**Conclusion:**

Utilizing a sonographer administered echo enhancement protocol results in reduced over 12 min of time saved per study.

## Introduction

From 2001 to 2011 the number of echocardiographic examinations in the United States increased 3.4% each year [1]. This number is anticipated to increase over the coming years as more individuals require medical care [2]. Echocardiography remains a versatile and valuable imaging modality in the care of cardiovascular patients; however, 10–20% of resting and 33% of stress echocardiographic examinations are determined to be suboptimal studies [3–5]. These suboptimal studies oftentimes lead to additional testing or changes in clinical management [5]. The American Society of Echocardiography (ASE) reports that about 75–90% of suboptimal echocardiographic examinations become interpretable with the use of an ultrasound enhancing agent (UEA) [6].

The ASE recommends the use of an UEA when two or more contiguous wall segments are not well visualized on non-contrast enhanced imaging [6–10]. Despite these recommendations, UEAs remain underutilized. In 2008, only 0.4% of all echocardiographic examinations performed in the United States utilized an UEA for left ventricular opacification, far below the rate of technically difficult studies [11]. This is despite the fact that the majority non-diagnostic echocardiograms can be made diagnostic with the use of UEA [12, 13]. A barrier to the use of an UEA is a perceived increase of resource utilization and cost related to the administration of contrast [14].

In 2013, Tang et al. published a study evaluating the feasibility of a sonographer administered UEA protocol in a large tertiary care facility [15]. Their results demonstrated an improvement in echo lab efficiency as well as an increase in productivity without any adverse effects. While the results of this study are noteworthy, only one sonographer was trained and not blinded to the study protocol. In June of 2015, a sonographer administered UEA protocol was initiated at our facility (level I trauma, tertiary referral center). Following a familiarization period, we sought to determine if institution of such a protocol achieved similar results when implemented on a larger scale with sonographers blinded to time tracking.

## Methods

One year prior to the study period, all sonographers employed at our facility were advised to maximize UEA use efficiency by carrying pre-activating perflutren lipid microsphere or perflutren protein-type A microspheres injectable suspension with them while performing portable echocardiographic examinations in the hospital’s wards and units. Sonographers were instructed to identify patients needing UEA early by either reviewing previous studies or assessing the need at the beginning of the study and informing the nurse in advance if an UEA was going to be needed. The decision to use an ultrasound contrast agent was made by the sonographer in real-time with a standing order to use contrast if two adjacent segments were not well visualized on apical images, the indication was for hypertrophic cardiomyopathy or ventricular thrombus in accordance with established ASE guidance; no physician input was necessary [6–9]. Sonographers would communicated directly with the patient’s nurse to have the nurse administer the UEA. Contrast was administered by the bolus method at the direction of the sonographer.

In June of 2015, four sonographers were selected to undergo specialized training in the indications, contraindications, preparation, administration, and monitoring of perflutren-based echo contrast agents. Training was completed in one day consisting of live didactics with hands on demonstration of the proper care and use of peripheral intravenous lines Sonographers administered contrast agent by bolus and would stop imaging to rebolus contrast as needed. The average years of experience between the four sonographers was fifteen with no sonographer having less than ten years of experience.

Following training, a sonographer administertion protocol was developed and approved by the hospital administration. The protocol covered only inpatient examinations obtained on weekdays (Monday through Friday) during standard business hours (0700–1700), thus all patient had intravenous access. The protocol implemented standing physician orders/authorization for patient selection and administration of perflutren-based echo contrast agents. Sonographers did not need to obtain permission to administer UEA. A physician with appropriate knowledge and experience of perflutren-based echo contrast agents was immediately available if a sonographer requested assistance. Inclusion criteria was the use of UEA during an echocardiogram performed on an inpatient, general wards and intensive care unit. Hospital policy is that all inpatients maintain intravenous access so the time needed to obtain intravenous access was removed as a confounder. Exclusion criteria were severe valvular heart disease requiring additional images as well, the use of strain or three dimensional imagining as it was felt this would contribute to total imaging time thus making the study appear to take longer.

Following a three month familiarization period in which sonographer administered contrast under the sonographer contrast administration protocol, a blinded three month observation period was started. This observation period of data was collected and compared to data obtained from the three months immediately prior to the training and implementation of the protocol. Sonographers were not made aware that imaging times were being recorded. During these observation periods all contrast enhanced studies performed by the study sonographer were identified and screened for exclusion criteria. All Echocardiograms included in the study were later independently reviewed by a level II or III echocardiographers who were blinded to the contrast protocol. The echocardiographer determined the appropriateness of contrast administration as well as the presence or absence of wall motion abnormalities on both the enhanced and non-enhanced images. The time to complete each portion of the study was determined by reviewing digital timestamps in the DICOM headers of each image. The total time to complete the study was determined by subtracting the time of first image stored from the time of the last image stored. The time to complete the pre-contrast images was the time of the last non-contrast image minus the time of the first non-contrast image. The transition time was the time of the first contrast image minus the time of the last non-contrast image. The time required to acquire the contrast images was the time from the first to the last contrast image.

Imaging was obtained using a Phillips® iE33 xMATRIX ultrasound system (Philips Healthcare, Bothell, WA) with a S5–1 phase array transducer (1.0–5.0 mHz). Sonographers used either perflutren lipid microsphere (Definitiy® Lantheus Medical Imaging Inc., Billerica, MA) or perflutren protein-type A microspheres injectable suspension (Optison™ GE Healthcare, Chicago, IL) when use of an UEA was clinically indicated. The decision to use either of the two perflutren-based contrast agents was made on product availability and administered according to manufacturer recommendations. Specifically perlutren protein-type A was via administration of 1 ml non diluted solution over 10 s, additional 1 ml re-boluses were administered as needed. Perflutren lipid microspheres were pre-activated at the beginning of the work day and diluted in 8.5 ml of saline, this was also administered as a 1 ml slow push with additional re-boluses as needed. Administration of both agents was followed by a saline flush. Imaging was interpreted by a level II or level III echocardiographer using a Merge Cardio™ (IBM Watson Health, Chicago, IL) platform. Echocardiogram interpretations were completed within the same day. Statistical analysis was performed using SPSS 21 (IBM Corporation, Armonk, New York). Chi-square test was used to categorical data and t-test was used for continuous variables.

## Results

A total of 320 inpatients (*n* = 63 pre-protocol, *n* = 257 post protocol) received a perflutren-based echo contrast agent during the study period. The median BMI of the pre and post protocol groups was 29.5 *kg*/*m*^2^ (range 19.5–55.1) and 29.1 *kg*/*m*^2^ (range 15.8–59.0) respectively (*p* = 0.09). The body surface area for the pre and post protocol groups was 2.1 *m*^2^ (range 1.5–2.8) and 2.0 (range 1.4–2.8) respectively (*p* = .14) (Table 1). The nurse administered protocol used more perflutren protein-type A microspheres (2.8 ml vs 2.4 ml, *p* = 0.03) than was used in the sonographer administration protocol. While the amount use of perflutren lipid microsphere was the same in both protocols (2.7 ml vs 3.1 ml, *p* = 0.22). The indication for echocardiographic examinations are presented in Table 2 and were not statistically significantly different (*p* = 0.375).

**Table 1:** Patient Characteristics and Contrast used. There was no difference in the body mass index, body surface area or amount of Definity used between the sonographer driven protocol (Pre) and following the protocol to allow sonographer administration of echocardiography image enhancer (Post). However, there was less Optison used in sonographer administration protocol, *p* = 0.03

	Pre	Post	*p* value
Body Mass Index (kg/m^2^)	29.5	29.1	0.09
Body Surface Area (m^2^)	2.1	2.0	0.14
Definity used (ml)	2.7	3.1	0.22
Optison used (ml)	2.8	2.4	0.03

**Table 2:** Echocardiogram Indications, breakdown of indications for echocardiograms prior to the sonographer driven protocol (Pre) and following the protocol to allow sonographer administration of echocardiography image enhancer (Post). No significant differences were noted, p = 0.375 by Chi-Squared

	Pre	Post
Arrhythmia/Abnormal ECG	8	16
Bacteremia/Endocarditis	4	5
Chest Pain/Dyspnea on Exertion	5	11
Edema	0	2
Abnormal Cardiovascular Exam finding	17	32
Heart Failure/Cardiomyopathy	6	10
Other	7	18
Pericardial disease	2	4
Stable Coronary Artery Disease	1	6
Syncope	3	0
TIA/CVA	4	3
Acute Coronary Syndrome	4	7
Valvular heart disease	0	1

**Fig. 1:**
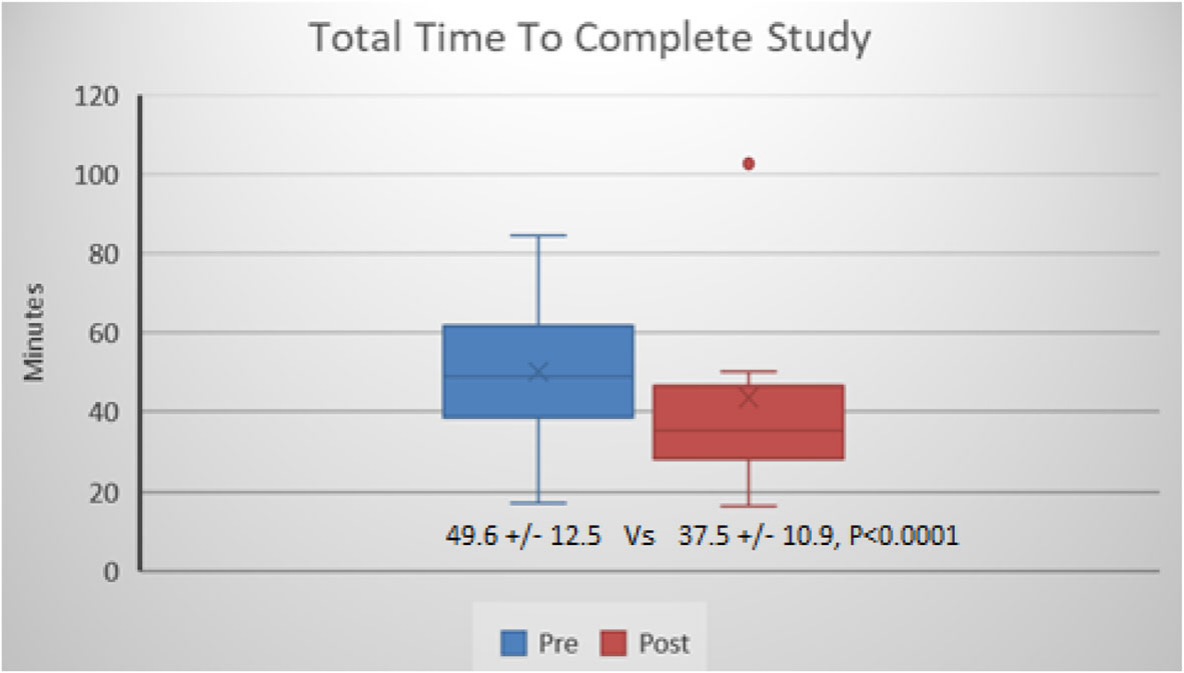
Total Time to Complete Study. Contrast enhanced echocardiograms required 49.6 min using a nurse administered contrast protocol compared to 37.5 min using a sonographer driven protocol, (*P* < 0.0001)

The median time to complete an echocardiographic examination (Fig. 1) to include time spent on contrast enhanced imaging was 49.6 ± 12.5 min in the pre-protocol group and 37.5 ± 10.9 min in the post protocol group (*p* < 0.0001). The post-protocol group acquired pre-contrast images in 27.7 ± 9.6 min versus 34.9 ± 12.4 min in the pre-protocol group. The mean time to transition from non-contrast to contrast images was 4:30 min faster in the post-protocol group (6.2 ± 3.8 min vs 10.8 ± 7.1minuted) compared to the pre-protocol echos. The time spent acquiring contrast enhanced imaging between the pre and post protocol groups were 4.0 ± 2.3 min v 3.8 ± 3.3 min (*p* = 0.68) respectively.

**Fig. 2:**
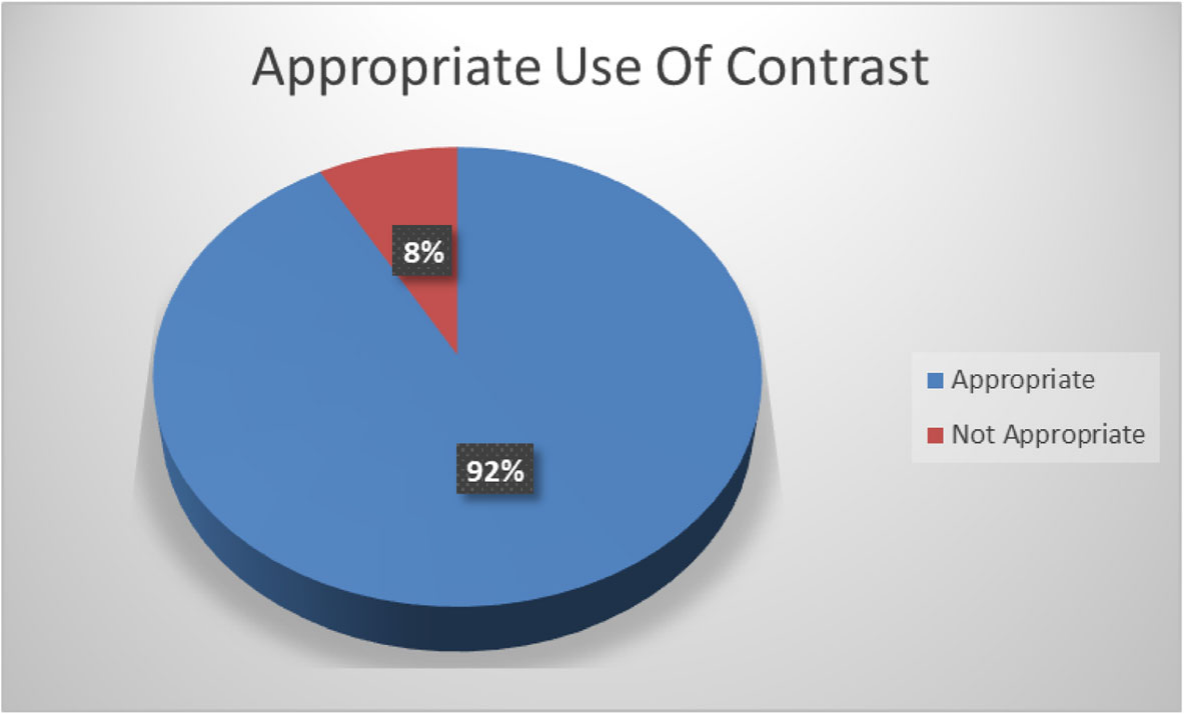
Appropriate Use of Contrast. Review of images by Cardiologist showed that 92% of studies receiving contrast in a sonographer driven protocol were appropriate

**Fig. 3:**
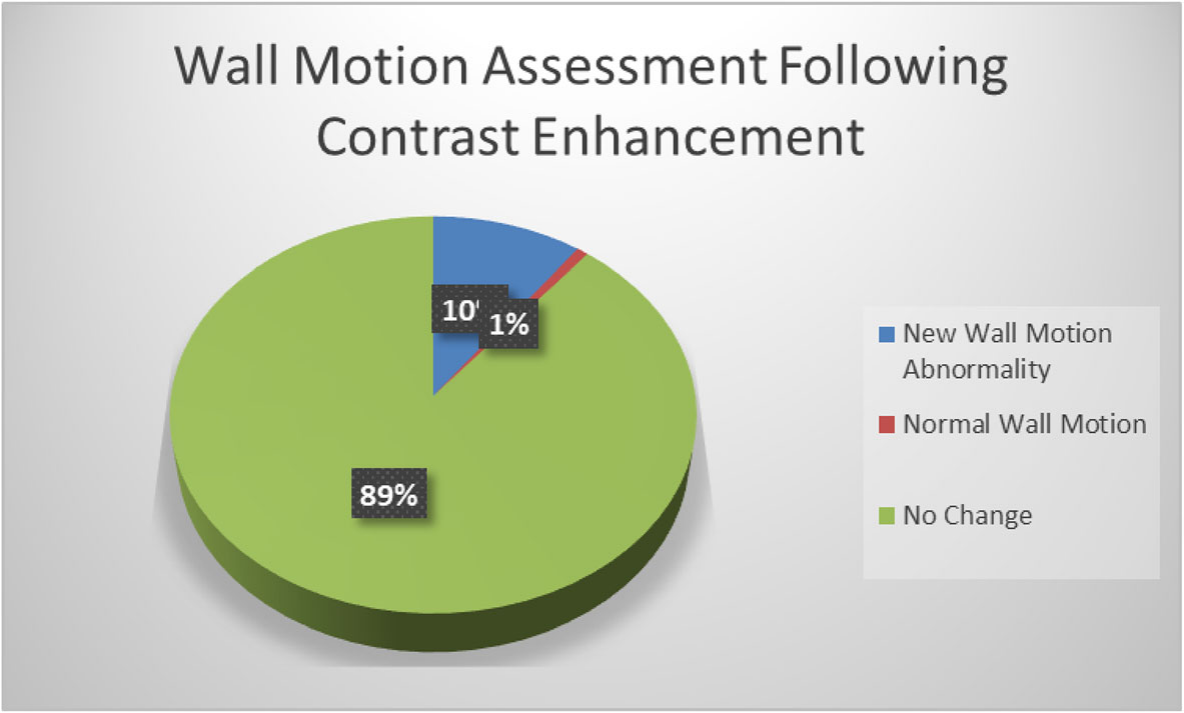
Wall Motion Assessment Following Contrast Enhancement. 10% of studies found a new regional wall motion abnormality on contrast images that was not seen on images without contrast. 1% found regional wall motion to be normal when unenhanced images suggested an abnormality. 89% had no change in the assessment of wall motion

Independent review of contrast enhanced examinations in the post protocol group were independently reviewed by a level II or level III echocardiographer. Of the 257 echocardiographic examinations performed following implementation of the protocol, 92% (*n* = 236) of examinations were determined to have utilized echo contrast in accordance with ASE guidelines (Fig. 2). The use of echo contrast resulted in a change in the wall motion assessment in 11% (*n* = 28) of patients with 10% (*n* = 26) of patients being identified as having a new wall motion abnormality after contrast enhancement. (Fig. 3). There were no adverse events from ultrasound enhancing agent in either of the administration protocols. No left ventricular thrombus or morphologic changes consistent with apical variant hypertrophic where found in the study echocardiograms.

## Discussion

Given the well-established benefits of contrast enhanced echocardiography, the American Society of Echocardiography (ASE) has called for programs and initiatives to be developed to better enable the sonographer in creating a proactive environment as it pertains to the use of echo contrast [6, 9, 14].

We demonstrated that initiation of a sonographer initiated, sonographer administered echo contrast protocol resulted in decreased time to complete each echocardiographic study by 12 min, 49.6 min to 37.5 min. A 7.2 min time savings was seen while non-enhanced images were being acquired, likely due to the lack of need for sonographer to stop acquiring non-enhanced images and notify a nurse that contrast will be needed. An additional 4.5 min of time savings was seen between the last non-enhanced image to first enhanced images. Time saving here could be from lack of need for sonographers to educated unfamiliar nursing staff to the use of UEAs and lack of need to wait for time pressed nurses to arrive to render assistance. Overall, there was a 12 min time savings from a sonographer driven protocol. This additional 12 min per study could result in enough time savings that one additional study could be performed over the course of a work shift. This 12 min time savings on the part of the sonographers does not take into account savings of nurse’s time. On a busy inpatient ward nurses’ time is at a premium. While similar results of improved efficiency have been reported in the literature [15], we are the first to report to our knowledge this improved efficiency in which the sonographers were blinded to monitoring intent. Moreover, our protocol did not require the presence nor the active approval of a physician to allow for contrast administration.

The use of echo contrast in the United States remains underutilized with a reported use in only 0.4% of all echocardiographic examinations in 2008 [13]. Suspected barriers limiting the utilization of echo contrast often relate to reimbursement, the Food and Drug Administration labeling, and the perceived decrement in workload efficiency as it pertains to contrast administration. However, over the last several years many noteworthy changes have been made.

In the United States, the use of echo contrast is reimbursed by Medicare as well as select private insurers for echocardiographic examinations obtained in the outpatient hospital or clinic setting [16]. However, inpatient echocardiography reimbursement remains based on Diagnostically Related Group (DRG) payment. While reimbursement may not be guaranteed, the downstream benefit is largely unrecognized.

In 2009 Kurt et al. reported that the use of contrast enhancement for suboptimal or technically limited studies conferred a net financial benefit, decreased the need for additional imaging, and improved the quality of care [7]. They report that the use of echo contrast resulted in 55% of patients in the surgical ICU, 31% of patients in the inpatient ward, and 33% of patients in the medical ICU no longer required additional testing following the use of echo contrast. The reason for additional testing revolved around the need for better delineation of left ventricular function and planned additional procedures included transthoracic echocardiogram (TTE) or nuclear perfusion imaging. In their cohort, the cost savings for not requiring additional procedures amounted to $122.0 per patient.

We found that in 11% of patients in the post protocol group, there was a change in wall motion assessment following the administration of echo contrast. Given the retrospective study design, we were not able to query providers to determine if the use of contrast had an impact on downstream patient management, but at the least, identifying new wall motion abnormalities gave the providers additional information to aid in patient care.

## Conclusions

Utilizing a sonographer driven protocol for selection and administration of perflutren-based echo contrast for inpatient TTE examinations resulted in reduced TTE study time by 12 min with an 11% improvement in wall motional assessment at our institution. As sonographer administered echo contrast protocols have shown to improve efficiency and increase productivity without compromising patient safety, more institutions should consider such implementation.

## Data Availability

The datasets during and/or analysed during the current study available from the corresponding author on reasonable request.

## Abbreviations

ASE: American Society of Echocardiography
UEA: Ultrasound enhancing agent
TTE: Transthoracic echocardiogram
